# Parents’ perception or children’s perception? Parental involvement and student engagement in Chinese middle schools

**DOI:** 10.3389/fpsyg.2022.977678

**Published:** 2022-11-17

**Authors:** Keqiao Liu, Yong Zhao, Miao Li, Wenjing Li, Yang Yang

**Affiliations:** ^1^School of Public Finance and Public Administration, Jiangxi University of Finance and Economics, Nanchang, China; ^2^Middle School Attached to Shandong University, Jinan, China; ^3^Department of Sociology, School of Philosophy and Social Development, Shandong University, Jinan, China; ^4^Center of Educational Technology, Institute of Advanced Studies in Humanities and Social Sciences, Beijing Normal University, Beijing, China

**Keywords:** parental involvement, student engagement, parents’ perception, children’s perception, middle school students, China

## Abstract

It is widely held that parental involvement plays a key role in enhancing student engagement, but less is known about whether and how parents’ and their children’s perceptions of different types of parental involvement relate to dimensions of student engagement, especially in the Chinese context. By surveying 2,219 students and their parents from nine middle schools in eastern China, this study found that only children’s perceptions of certain types of parental involvement (e.g., parent–child communication), rather than those of their parents, correlated with student engagement (i.e., behavioral engagement, emotional engagement, and cognitive engagement). Further, different types of parental involvement presented varied relationships with dimensions of student engagement. This study deepens our understanding of the dynamic interplay between parental involvement and student engagement in view of parents’ and children’s perceptions.

## Introduction

Many studies view student engagement as an antecedent of students’ academic success by shielding students from educational risks (e.g., dropping out of school) and assisting them to perform better (Finn and Rock, 1997; Fredricks et al., 2004; Lee, 2014; Schnitzler et al., 2020). On the other hand, parental involvement plays a vital role in enhancing school engagement among students (Connell et al., 1994; You and Sharkey, 2009; Al-Alwan, 2014; Yang et al., 2021). This potential important impact of parental involvement may vary depending on whether parental involvement is perceived by parents or their children in that prior research detect discrepancies between parents’ and their children’s perceptions of parental involvement (Paulson and Sputa, 1996; DePlanty et al., 2007; Thomas et al., 2020; Liu et al., 2021). However, there is currently a lack of research on the associations between parents’ and their children’s perceptions of parental involvement and student engagement. Understanding whose perception of parental involvement matters more on student engagement can (1) assist future researchers in determining whose perception of parental involvement to examine in their research, (2) allow professionals to facilitate student engagement more effectively, and (3) help parents and related others to recognize the parent–child relationship problems that may be masked by the perception differences in parental involvement as suggested by Korelitz and Garber (2016). Therefore, considering the aforementioned, this study examined the relationship between parental involvement and engagement from the perspectives of parents and their children.

When examining the relationship between parental involvement and student engagement, few studies have incorporated the multiple dimensions of student engagement (Connell et al., 1994; You and Sharkey, 2009; Yang et al., 2021), and thus these analyses weaken the explanatory power of the findings and implications. This study adopted the three dimensions of student engagement defined by Fredricks et al. (2004), which are behavioral, emotional (or affective), and cognitive engagement. Specifically, behavioral engagement refers to students’ positive conduct, involvement, and participation in academic-related tasks and activities. Emotional engagement measures students’ sense of belonging, valuing, and positive emotional reactions toward their school and school members. Lastly, cognitive engagement conveys students’ psychological and strategic investment in learning activities (Fredricks et al., 2004, 2016).

When it comes to the measurement of parent involvement, previous studies either consider it as a unidimensional construct or merely examine its one or two types (Connell et al., 1994; Fan and Chen, 2001; Jeynes, 2007; Al-Alwan, 2014), which calls for a thorough investigation on parental involvement. In response to the urgent demand, this study defined parental involvement as parents’ participation in children’s education and parents’ experiences with their children (Jeynes, 2007; Liu et al., 2021). Informed by the work of Hill and Tyson (2009) who also focused on middle school students, this study distinguished two types of parental involvement—home-based involvement and school-based involvement. Specifically, the home-based involvement includes parental academic involvement, parental daily involvement, and parent–child communication; and the school-based involvement was measured by parental school participation.

This study examined parents’ and children’s perceptions of parental involvement and their associations with student engagement in nine middle schools in eastern China. Two research questions guided the present inquiry:

1. Are there any differences between parents’ and their children’s perceptions of parental involvement in the context of Chinese middle schools?
2. Considering the incongruence between parents’ and their children’s perceptions of parental involvement, what are the relationships between different types of parental involvement and the three dimensions of student engagement?

## Study framework

**FIGURE 1:**
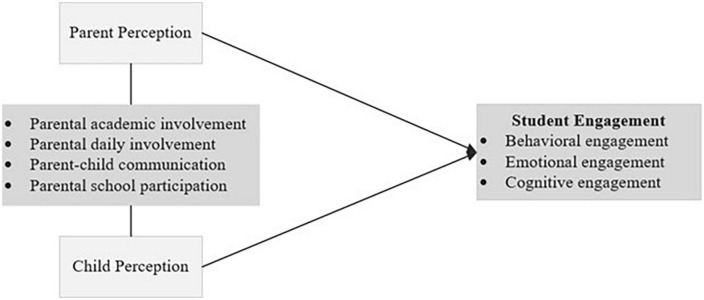
Study framework guiding the present study.

The research questions were set in the framework shown in Figure 1. Because parents and their children can hold different perceptions of parental involvement (Paulson and Sputa, 1996; DePlanty et al., 2007; Thomas et al., 2020), perception discrepancies in parental involvement might yield different impacts on the dimensions of children’s engagement. On the other hand, a recent study illustrates that, compared with their parents, children’s perceptions of parental involvement have a closer relationship with their academic achievement (Thomas et al., 2020). Epstein’s (1995) model of school, family, and community partnerships offered a valuable insight. Specifically, students rather than the related others played an essential role in their own education, development, and success at school. The following literature review focused on (1) perception differences in parental involvement between parents and their children; and (2) the relationships between types of parental involvement and dimensions of student engagement.

## Literature review

### Differences in parents’ and their children’s perceptions of parental involvement

Discrepancies exist between parents’ and their children’s perceptions of parental involvement (DePlanty et al., 2007; Thomas et al., 2020; Liu et al., 2021; Yang et al., 2021). Some researchers indicate that parents, on average, report a higher level of parental involvement than their children (e.g., Paulson and Sputa, 1996; DePlanty et al., 2007). For example, DePlanty et al. (2007) have found that parents report a higher level of parental involvement behaviors (e.g., talking to teachers) than their children. In contrast, Liu et al. (2021) have found that parents report a lower level of parental involvement (i.e., parental academic involvement and parent–teacher communication) than their children. Nonetheless, previous studies were mostly confined to Western developed countries. Considering the cultural differences in raising children (Lui and Rollock, 2013; Fu and Markus, 2014), parents’ and their children’s perceptions of parental involvement in Chinese families might show different patterns. Compared with the few relevant studies on Chinese students conducted at the outset of COVID-19 when the stay-at-home order was in place (Liu et al., 2021; Yang et al., 2021), the current study examined parents’ and their children’s perceptions of parental involvement after schools reopened and may yield different results.

Among the studies examining the effect of different perceptions of parental involvement, children’s perception of parental involvement generally matters more than parents’ perception on children’s academic achievement and mental wellbeing (Thomas et al., 2020; Liu et al., 2021; Yang et al., 2021). For example, Liu et al.’s (2021) study shows that children’s perception of parental academic involvement and children’s perception of parent–child communication rather than their parents’ perceptions are associated with children’s depression tendency. Similar to Paulson and Sputa’s (1996) conjecture, the findings suggest that the perceptions of parental involvement weigh more than the actual parental involvement behaviors in predicting children’s academic achievement and wellbeing. This can be explained by Epstein (1995), p. 702 model of school, family, and community partnerships, which regards children as “the main actors in their education, development, and success in school.” Thus, it is when children perceive their parents involving in their education and development, their engagement could be influenced.

### Parental involvement and children’s engagement

#### Behavioral engagement

The extant studies support a positive relationship between parental involvement and students’ behavioral engagement. For instance, more frequent communications between parents and children (You and Sharkey, 2009; Wang and Sheikh-Khalil, 2014) and parents who spend more time on their children’s study and cultivate an enriching learning environment (Wang and Sheikh-Khalil, 2014) are related to students’ higher level of behavioral engagement. Similarly, in a more general sense, a higher level of parental involvement is associated with a higher level of behavioral engagement, where the magnitude of this relationship is larger than the magnitude of the positive associations among parental involvement and students’ emotional and cognitive engagement (Al-Alwan, 2014).

In contrast, other studies suggest that parental involvement might not relate to or even negatively relate to students’ behavioral engagement. Prior parental involvement studies on students’ academic performance support this argumentation. For example, according to a literature review, several studies on students of different racial/ethnic groups (e.g., Mexican American and Korean students) find that parents’ school-based involvement is not associated with students’ academic performance (Boonk et al., 2018). However, some other studies on students of different countries (e.g., Spanish and US students) indicate that a higher level of parental school participation (Chowa et al., 2013), a higher level of parental control (Nunez et al., 2015; Erdem and Kaya, 2020), and having a parent who checked homework (Desimone, 1999) are related to a lower level of students’ academic performance. Considering the positive relationship between students’ behavioral engagement and their academic performance that past studies index (e.g., Fredricks et al., 2004; Lei et al., 2018), it can be assumed that not all aspects of parental involvement behaviors would yield positive effects on students’ behavioral engagement. Some studies supporting this proposition reveal that school-based parental involvement is not related to students’ behavioral engagement (Wang and Sheikh-Khalil, 2014).

Except for those studies which neglect the different dimensions of student engagement (e.g., Shernoff et al., 2016), it is worth noting that most extant studies focus on students’ behavioral engagement, rather than emotional and cognitive engagement (Fredricks et al., 2004; Salmela-Aro et al., 2021). Most strikingly, few studies have examined the relationships between different types of parental involvement and behavioral engagement of the students. Even less research has explored the potential effects of varied parental involvement behaviors on different dimensions of student engagement, not to mention the incorporation of the perceptions of both parents and their children.

#### Emotional engagement

Previous studies demonstrate that a higher level of parental involvement is associated with a higher level of students’ emotional engagement. For example, more frequent communication between parents and children (Yang et al., 2021) and a higher level of school-based involvement (Wang and Sheikh-Khalil, 2014) are associated with a higher level of students’ emotional engagement. Meanwhile, among studies that did not differentiate different types of parental involvement, parental involvement is positively related to students’ emotional engagement (Al-Alwan, 2014; Xiong et al., 2021).

In contrast, some research confirms that parental involvement is not associated with or even negatively associated with students’ emotional engagement. This proposition could be implied by the non-existent or negative relationships between aspects of parental involvement and students’ academic performance (e.g., Chowa et al., 2013; Boonk et al., 2018), because emotional engagement is positively associated with academic performance (Lei et al., 2018). The study of Ansong et al. (2017) corroborates the proposition on the potential non-positive relationship between parental involvement and emotional engagement, in which a higher level of parental involvement correlates with a lower level of students’ emotional engagement.

To wrap up, this study has three main conclusions. First, few studies have examined the relationships between different types of parental involvement and students’ emotional engagement. Second, there is an unresolved conundrum regarding how aspects of parental involvement are associated with children’s emotional engagement. For example, parents’ school-based involvement might be not related, negatively related, or positively related to emotional engagement. These discrepancies in conclusions could be traced to the fact that scholars measure different types of parental involvement and utilize varying definitions and classifications of emotional engagement. In the meantime, some studies fail to discern different types of parental involvement (e.g., Ansong et al., 2017) and hence conceal the variations within parental involvement. Lastly, the current scholarship entails valuing the perspectives of parents and their children, because both are important stakeholders in making sense of parental involvement.

#### Cognitive engagement

Similar to behavioral and emotional engagement, parental involvement is widely regarded as being positively associated with students’ cognitive engagement. Marshall and Jackman (2015) find that a higher level of parental involvement in their children’s homework is related to a higher level of students’ cognitive engagement, although this relationship might be mediated by students’ autonomous motivation for doing homework (Nunez et al., 2019). Meanwhile, Fan and Williams (2010) indicate that parents who participate in activities with their children and who communicate with school authorities tend to have children with a higher level of cognitive engagement.

In contrast, parental involvement might be either not associated with or negatively associated with students’ cognitive engagement. The potential none or negative relationships between aspects of parental involvement and students’ academic performance (e.g., Boonk et al., 2018) could imply the possible none or negative relationships between aspects of parental involvement and students’ cognitive engagement. Some scholars maintain that cognitive engagement and academic performance are positively related to each other (Lei et al., 2018; Xie et al., 2020). Similarly, in the research conducted by Fan and Williams (2010), several parental involvement factors, such as parental participation in school functions, are not related to students’ cognitive engagement. Further, their study suggests that parent–school communication could be negatively associated with students’ cognitive engagement, especially when such communications pertain to students’ poor academic performance.

Notably, cognitive engagement remains an under-examined dimension of student engagement (Fredricks et al., 2004; Salmela-Aro et al., 2021), because few studies have scrutinized whether and how parental involvement could affect cognitive engagement. Furthermore, none have examined the effects of parental involvement on cognitive engagement by drawing upon the perceptions of parents and their children. Also, the extant studies diverge on the definitions and classifications of parental involvement and cognitive engagement, leaving these findings uncomparable with one another (e.g., Fan and Williams, 2010; Marshall and Jackman, 2015). In a nutshell, the review on the relationship between parental involvement and student engagement suggests that variations in the effects of parental involvement are evident both within and among the three dimensions of student engagement.

## Materials and methods

### Participants and procedures

Using convenience sampling, this study surveyed students and their parents from nine urban middle schools that were affiliated to a prestigious university in eastern China. The surveyed middle schools provided formal education from seventh to ninth grade, where most students aged around 12–14 years at the time of this study (Mean = 13.202, *SD* = 0.904, Min = 11, Max = 16). The corresponding author of this study worked as a faculty member at the university, which offered us the opportunity to access those schools. We collected the data using Wenjuanxing (a popular survey platform in China) between November 27, 2020 and January 6, 2021 (the 2020 fall semester ended around January 25, 2021). At the time of this survey, the COVID-19 situation in China was generally stable, where schools were largely back to normal. The sampled students filled in the student questionnaire on school computers under the supervision of teachers who were trained to follow the steps on the information sheet provided by the research team. Meanwhile, we asked students to bring back home an information sheet to their parents. On the parents’ sheet, besides a brief introduction, we attached a QR code and a link of the parents’ questionnaire, with which parents can decide to fill out their questionnaire using a personal computer or a mobile device. We later matched the questionnaires of children and their parents by linking variables. Participation in the study was voluntary and anonymous, and the participants can withdraw from the study at any time. This study was approved by the Academic Committee of Shandong University to protect the rights of the research participants.

Ultimately, we collected data from 2,219 students, with linked parental data (*N*_parent_ = 2,219). Since the nine middle schools under study catered to 7,512 students (as reported by the schools), approximately 29.5% of the students completed the students’ questionnaire. 52.3% of the surveyed students were females (vs. 47.7% males) and 96.8% of them were Han (the majority ethnic group in China). The estimated average annual family income of the sample was 34,056 USD (as reported by the parents), while the average annual income of employed people in 2020 was approximately 15,060 USD in urban areas of China (National Bureau of Statistics of China, 2021).

Since the online questionnaires were used, we were able to adopt the forced answering option, though respondents could choose not to start the survey or drop out. This led to no missing values in the data.

### Variables

We designed the students’ and parents’ questionnaires based on extant instruments and relevant research. Besides, we consulted experts in the field of educational psychology and included suggestions on the types of parental involvement. We also discussed the questionnaires with school administrators and teachers in the surveyed schools. Since the questionnaires were distributed in Chinese, members of the research team first translated the related items from English to Chinese, then two other team members back-translated the items. To reach a final version of the questionnaires, discrepancies were discussed and solved by the research team. Before the survey, we carried out a pilot study. The aim was to ensure the validity and reliability of the questionnaires and to make sure that the target schools permitted us to distribute the questionnaires.

#### Dependent variable

##### Student engagement

It consisted of behavioral engagement, emotional engagement, and cognitive engagement. Since, for example, students’ homework behaviors (e.g., time and effort spent on homework) in one academic subject (e.g., English) can positively and significantly relate to their homework behaviors in another academic subject (e.g., math) (Trautwein et al., 2006), we did not measure student engagement in different academic subjects. Similarly, some existent quantitative studies tend to measure student engagement in a more general sense rather than focus on different academic subjects (e.g., Wang and Sheikh-Khalil, 2014; Nunez et al., 2015); however, we do admit that student engagement can vary across different academic subjects, for which future related studies that consider different academic subjects may be needed.

We assessed behavioral engagement with six items, such as the question that asked: “during the past 2–3 months, I complete homework on time,” with answers on a 5-point scale (1 = never, 2 = a few times, 3 = sometimes, 4 = often, 5 = always). The items originated from the Student Participation Questionnaire (SPQ) (Finn et al., 1995; Finn and Zimmer, 2012), which asked teachers to rate their students. The SPQ contained 4 subscales on “effort,” “initiative,” “non-participatory behavior,” and “value” respectively (Finn and Zimmer, 2012). Based on the definition of behavioral engagement that we adopted in this study, we selected six items from the “effort” subscale and then converted them into items for students to answer. An exploratory factor analysis detected one dimension (behavioral engagement), and the reliability coefficient was acceptable (Cronbach’s α = 0.786). To compare behavioral engagement with the other two types of student engagement without the inclination to calculate standardized scores, we created a derived variable based on the mean rather than the sum of items, with a higher value indicating a higher level of student behavioral engagement (mean = 4.546, *sd* = 0.506).

We measured emotional engagement with five items. For example, one item asked: “I think that school is important,” with replies on a 5-point scale (1 = never, 2 = a few times, 3 = sometimes, 4 = often, 5 = always). We adapted items from the SPQ “value” subscale. We also added items such as “I am proud of being a part of this school” based on the Identification with School Questionnaire (Voelkl, 1996) and the study of Finn and Zimmer (2012). An exploratory factor analysis found one dimension (emotional engagement), and the reliability coefficient was acceptable (Cronbach’s α = 0.875). We created a derived variable based on the mean of the items, with a higher value indicating a higher level of emotional engagement (mean = 4.551, *sd* = 0.653).

We assessed cognitive engagement with 11 items. For instance, one item asked: “I attempt to do my schoolwork thoroughly and well, rather than just trying to get by,” with answers on a 5-point scale (1 = never, 2 = a few times, 3 = sometimes, 4 = often, 5 = always). We selected and adapted these items from the “effort” and “initiative” subscales of the SPQ (Finn and Zimmer, 2012). The exploratory factor analysis extracted one dimension (cognitive engagement), and the reliability coefficient was acceptable (Cronbach’s α = 0.933). We created a derived variable based on the mean of the items, with a higher value indicating a higher level of cognitive engagement (mean = 4.014, *sd* = 0.824).

Beside of the exploratory factor analyses, we ran a three-factor confirmatory factor analysis. The standardized factor loadings for behavioral engagement ranged from 0.521 to 0.718 (4 out of 6 loadings around/above 0.700), for emotional engagement ranged from 0.724 to 0.843, for cognitive engagement ranged from 0.652 to 0.868 (8 out of 11 loadings around/above 0.700). The Comparative Fit Index (CFI) = 0.866 (good fit), the Standardized Root-Mean-Square Residual (SRMR) = 0.066 (good fit), and the Root-Mean-Square Error of Approximation (RMSEA) = 0.095 (marginal fit), which suggested acceptable fit of our model (Browne and Cudeck, 1993; Bong et al., 2013; Dagnall et al., 2018).

#### Independent variables

##### Home-based parental involvement

It included parental academic involvement, parental daily involvement, and parent–child communication. We collected information on both parents and their children’s perceptions of these types of parental involvement. We designed the measurement of parental academic involvement based on the type of parental involvement about “learning at home” identified by Epstein (1995), who defined it as homework and other academic-related help that parents provided to their children at home.

Parents’ perception of academic involvement included two items. Specifically, they were “I help my child to do his/her homework” (Homework Help) and “I check my child’s homework” (Homework Check), with replies on a 4-point scale. The mean for Homework Help was 2.880 (*sd* = 0.871), while the mean for Homework Check was 3.160 (*sd* = 0.852). We measured child perception of parental academic involvement with the same items as those of their parents, but we redrafted these items to tailor to the parents’ stance. The mean for Homework Help was 2.980 (*sd* = 1.009), and the mean for Homework Check was 3.050 (*sd* = 1.024).

In line with the study of Kim et al. (2016), we viewed parental daily involvement as the aspect of parental involvement that “support[ed] non-academic development” of children. It measured non-academic related support that parents offered to their children. Contrary to their study, however, we separated parent–child communication from parental daily involvement in this study instead of lumping the two as representations of “non-academic development.” We measured parental perception of parental daily involvement with three items. For example, “I support my child to participate in extracurricular activities,” with answers on a 4-point Likert scale. An exploratory factor analysis found one dimension (parental daily involvement), and the reliability coefficient was acceptable (Cronbach’s α = 0.859). We created a derived variable based on the mean of the items, with a higher value indicating a higher level of parental daily involvement perceived by parents (mean = 3.545, *sd* = 0.503). Child perception of parental daily involvement consisted of the same items. An exploratory factor analysis extracted one dimension (parental daily involvement), and the reliability coefficient was acceptable (Cronbach’s α = 0.822). We created a derived variable based on the mean, with a higher value indicating a higher level of parental daily involvement perceived by children (mean = 3.504, *sd* = 0.621).

We employed the definition of parent–child communication offered by Davidson and Cardemil (2009), meaning the exchange of factual and emotional information between parents and children. We also considered the instrument in the study of Liu et al. (2021) which adapted Krohn et al.’s (1992) communication scale. Parental perception of parent-child communication was examined by six items, including the question that “my child talks about things that bother him/her,” with replies on a 4-point Likert scale. An exploratory factor analysis detected one dimension (parent–child communication), and the reliability coefficient was acceptable (Cronbach’s α = 0.923). We created a derived variable based on the mean of items, with a higher value indicating a higher level of parent–child communication perceived by parents (mean = 3.146, *sd* = 0.616). We measured child perception of parent–child communication using the same items as those of parents. An exploratory factor analysis extracted one dimension (parent–child communication), and the reliability was acceptable (Cronbach’s α = 0.922). We created a derived variable based on the mean, with a higher value indicating a higher level of parent–child communication perceived by children (mean = 3.259, *sd* = 0.738).

Beside of the exploratory factor analyses, we ran two two-factor confirmatory factor analyses. The standardized factor loadings for parental perception of parental daily involvement ranged from 0.737 to 0.865 and for parental perception of parent–child communication ranged from 0.710 to 0.898. The CFI = 0.949 (good fit), the SRMR = 0.056 (good fit), and the RMSEA = 0.111 (poor fit). Although the RMSEA index did neither suggest good fit nor marginal fit, we still kept the items based on the other indices and the results of child perception. Specifically, the standardized factor loadings for child perception of parental daily involvement ranged from 0.751 to 0.817 and for child perception of parent–child communication ranged from 0.719 to 0.873. The CFI = 0.958 (good fit), the SRMR = 0.034 (good fit), and the RMSEA = 0.097 (marginal fit), which suggested acceptable fit of our model (Browne and Cudeck, 1993; Bong et al., 2013; Dagnall et al., 2018).

##### School-based parental involvement

In this study, the definition of parental school participation originated from the type of parental involvement pertaining to “volunteering” (Epstein, 1995). Parental school participation described the action of parents who provided support and helped in school events and activities. Parental perception of parental school participation consisted of two items. Specifically, the items were “I help to organize activities and events at my child’s school” (Help) and “I attend activities and events held by my child’s school” (Attend), with answers on a 4-point scale. The mean for Help was 3.140 (*sd* = 0.833), and the mean for Attend was 3.380 (*sd* = 0.701). Child perception of parental school participation focused on the same participation behaviors. The mean for Help was 3.240 (*sd* = 0.888), and the mean for Attend was 3.440 (*sd* = 0.805).

#### Control variables

When examining the relationships between parental involvement variables and student engagement variables, we controlled student and parent background variables, including student gender (52.3% females) (student report), whether a student being the only child (44.1% only child) (parent report), student ethnicity (96.8% Han vs. 3.2% other minorities) (student report), grade level (39.5% grade 7, 31.1% grade 8, and 29.4% grade 9) (student report), family annual income in USD (mean = 3.406, *sd* = 3.151) (parent report), and parents’ educational level (66.3% mother and 68.9% father received postsecondary education) (parent report). The detailed variable descriptions are available in the Supplementary Appendix A.

### Data analysis

Using SPSS 26, we conducted both descriptive and inferential analyses. To answer research question 1, we compared the means of variables and ran simple correlations among the parental involvement variables. To answer research question 2, without controlling for any other effects, we ran simple correlations among parental involvement variables and student engagement variables. Next, we carried out multiple linear regression analyses with school fixed effects in two steps. Step 1 contained only parental involvement variables and school fixed effects. Step 2 added the control variables (e.g., students’ gender) to the Step 1 model. A comparison between the two models allowed us to examine changes in the effects of parental involvement variables on student engagement.

## Results

### Parents’ and their children’s perceptions of parental involvement

Table 1 shows that within parental academic involvement, parents’ perception of Homework Help was lower than their children’s perception (paired samples *t*-test: Mean Gap = –0.100, *p* < 0.05), while parents’ perception of Homework Check was higher than their children’s perception (paired samples *t*-test: Mean Gap = 0.110, *p* < 0.05). The means for Homework Check were higher than the means for Homework Help. In other words, both parents and children perceived a higher level of Homework Check than Homework Help. Meantime, parents’ perception of parental daily involvement was generally higher than their children’s perception (paired samples *t*-test: Mean Gap = 0.041, *p* < 0.05). In contrast, parents’ perceptions of parent–child communication (paired samples *t*-test: Mean Gap = –0.113, *p* < 0.05) and parental school participation (including Help and Attend) (paired samples *t*-test: Mean Gap_help_ = –0.100, *p* < 0.05; paired samples *t*-test: Mean Gap_attend_ = –0.060, *p* < 0.05) were lower than children’s perception. A comparison between the parental involvement variables indicated that both parents and their children perceive a higher level of parental daily involvement than the other aspects of parental involvement. Meanwhile, both parents and children perceived a lower level of Homework Help (belonging to parental academic involvement) than the other aspects of parental involvement.

**TABLE 1:** Parents’ versus students’ perceptions on parental involvement.

	Parent report		Student report		Gap^a^
	Mean	*SD*	Mean	*SD*	(Parent–student)
**Parental academic involvement**					
Homework help	2.880	0.871	2.980	1.009	-0.100***
Homework check	3.160	0.852	3.050	1.024	0.110***
Parental daily involvement	3.545	0.503	3.504	0.621	0.041**
Parent–child communication	3.146	0.616	3.259	0.737	-0.113***
**Parental school participation**					
Help	3.140	0.833	3.240	0.888	-0.100***
Attend	3.380	0.701	3.440	0.805	-0.060**

^a^Paired samples *t*-tests were conducted.

*p < 0.05, **p < 0.01, and ***p < 0.001.

Table 2 presents correlations among the parental involvement variables. We found positive and moderate relationships between parents’ perception of Homework Help and their perception of Homework Check (*r* = 0.511, *p* < 0.05), between children’s perception of Homework Help and their perception of Homework Check (*r* = 0.632, *p* < 0.05), between children’s perception of parental daily involvement and their perception of parent–child communication (*r* = 0.513, *p* < 0.05), between parents’ perception of Help and their perception of Attend (*r* = 0.645, *p* < 0.05), and between children’s perception of Help and their perception of Attend (*r* = 0.647, *p* < 0.05). However, other correlations among parental involvement variables presented none to weak relationships. For example, parents’ perception of parental daily involvement was positively but weakly associated with that of their children’s perception (*r* = 0.214, *p* < 0.05).

**TABLE 2:** Correlations among parental involvement variables.

		1	2	3	4	5	6	7	8	9	10	11
1	Homework help (parent report)	1										
2	Homework Help (student report)	0.376										
3	Homework check (parent report)	0.511	0.219									
4	Homework check (student report)	0.222	0.632	0.311								
5	Parental daily involvement (parent report)	0.159	0.097	0.144	0.084							
6	Parental daily involvement (student report)	0.107	0.284	0.058	0.217	0.214						
7	Parent–child communication (parent report)	0.196	0.065	0.212	0.043	0.383	0.169					
8	Parent–child communication (student report)	0.111	0.344	0.075	0.302	0.163	0.513	0.316				
9	Help (parent report)	0.224	0.066	0.194	0.077	0.196	0.096	0.255	0.098			
10	Help (student report)	0.104	0.302	0.089	0.304	0.149	0.360	0.104	0.311	0.313		
11	Attend (parent report)	0.162	0.044	0.145	0.071	0.230	0.089	0.264	0.090	0.645	0.239	
12	Attend (student report)	0.042	0.228	0.039	0.261	0.145	0.327	0.109	0.320	0.219	0.647	0.241

(1) Areas in gray indicate correlations ≥ 0.500; (2) All the correlations were statistically significant except for the relationship between Homework Check (parent report) and Attend (student report).

### Correlations between parental involvement and student engagement

All parental involvement variables were positively associated with student behavioral engagement, even though the magnitudes of the relationships ranged from none to weak (see Table 3). Children’s perception of parental daily involvement (*r* = 0.374, *p* < 0.05) and their perception of parent-child communication (*r* = 0.372, *p* < 0.05) presented the strongest associations with behavioral engagement. In contrast, parents’ perception of parental academic involvement (Homework Help: *r* = 0.086, *p* < 0.05; Homework Check: *r* = 0.047, *p* < 0.05) and their perception of parental school participation (Help: *r* = 0.087, *p* < 0.05; Attend: *r* = 0.063, *p* < 0.05) tended to dissociate with behavioral engagement.

**TABLE 3:** Correlations between parental involvement variables and student school engagement.

	Behavioral engagement	Emotional engagement	Cognitive engagement	VIF
Behavioral engagement	1			
Emotional engagement	0.577			
Cognitive engagement	0.693	0.586		
Homework help (parent report)	0.086	0.100	0.094	1.592
Homework help (student report)	0.204	0.200	0.253	2.011
Homework check (parent report)	0.047	0.038	0.047	1.504
Homework help (student report)	0.175	0.175	0.248	1.875
Parental daily involvement (parent report)	0.128	0.143	0.171	1.239
Parental daily involvement (student report)	0.374	0.393	0.398	1.493
Parent–child communication (parent report)	0.134	0.138	0.139	1.391
Parent–child communication (student report)	0.372	0.417	0.447	1.628
Help (parent report)	0.087	0.080	0.119	1.872
Help (student report)	0.238	0.258	0.376	1.974
Attend (parent report)	0.063	0.066	0.083	1.795
Attend (student report)	0.228	0.254	0.337	1.826

(1) Areas in gray indicate correlations ≥ 0.500; (2) All the correlations were statistically significant except for the relationship between Homework Check (parent report) and emotional engagement.

Parental involvement variables exhibited positive relationships with student emotional engagement, even though the magnitudes of the associations ranged from none to weak (see Table 3). Children’s perception of parent–child communication (*r* = 0.417, *p* < 0.05) and their perception of parental daily involvement (*r* = 0.393, *p* < 0.05) had the strongest relationships with emotional engagement. In contrast, parents’ perception of parental academic involvement (Homework Help: *r* = 0.100, *p* < 0.05; Homework Check: *r* = 0.038, *p* > 0.05) and their perception of parental school participation (Help: *r* = 0.080, *p* < 0.05; Attend: *r* = 0.066, *p* < 0.05) tended to disconnect with emotional engagement.

Similarly, all parental involvement variables were positively associated with student cognitive engagement, while the magnitudes of the associations ranged from none to weak (Table 3). Children’s perception of parent–child communication (*r* = 0.447, *p* < 0.05), of parental daily involvement (*r* = 0.398, *p* < 0.05), and their perception of parental school participation (Help: *r* = 0.376, *p* < 0.05; Attend: *r* = 0.337, *p* < 0.05) had the strongest relationships with cognitive engagement. In contrast, parents’ perception of parental academic involvement (Homework Help: *r* = 0.094, *p* < 0.05; Homework Check: *r* = 0.047, *p* < 0.05) and their perception of parental school participation (Help: *r* = 0.119, *p* < 0.05; Attend: *r* = 0.083, *p* < 0.05) tended to dissociate with cognitive engagement.

In general, children’s perception of parental daily involvement and their perception of parent-child communication had the strongest relationships with the three dimensions of student engagement, while parents’ perception of parental academic involvement and their perception of parental school participation presented the weakest associations with student engagement. Notably, children’s perception of parental school participation had a stronger relationship with student cognitive engagement than with behavioral engagement and emotional engagement. The findings suggested that students’ perceptions of parental involvement behaviors matter more on student engagement than their parents’ perceptions. Although similarities exist in the effects of parental involvement behaviors on the three dimensions of student engagement, disparities can be detected.

### Multiple linear regression models predicting student engagement

As demonstrated in Table 2, none of the relationships among parental involvement variables had values that were equal to or greater than |0.7|, suggesting the non-existence of collinearity among the parental involvement variables. In addition, because none of the Variance Inflation Factors (VIFs) for parental involvement variables were > 5 (Table 3), we detected no multicollinearity. Thus, it is reasonable to include both parents’ and their children’s perceptions of parental involvement in the same regression models.

It is worth noting that the parental academic involvement (i.e., Homework Help and Homework Check) and parental school participation (i.e., Help and Attend) variables were measured by a single item on a 4-point scale, for which we treated as continuous variables. This may harm the interpretations of our results. Accordingly, we also ran multiple linear regression analyses by dummy coding the aforementioned variables (0 = never and a few times, 1 = sometimes and always) (Supplementary Appendix B). A comparison of the results in Table 4 and Supplementary Appendix B showed similarities. In this study, we focused on the results of Table 4, though we suggested future studies to utilize more items to measure parental academic involvement and parental school participation to ensure the validity of these two variables.

**TABLE 4:** Stepwise multiple linear regressions with school fixed effects.

	Behavioral engagement		Emotional engagement		Cognitive engagement	
	Model 1	Model 2	Model 1	Model 2	Model 1	Model 2
	*B* (*se*)	*B* (*se*)	*B* (*se*)	*B* (*se*)	*B* (*se*)	*B* (*se*)
**Parental academic involvement (parent report)**						
Homework help	–0.006 (0.014)	–0.010 (0.014)	0.022 (0.018)	0.022 (0.018)	–0.003 (0.021)	–0.005 (0.021)
Homework check	0.001 (0.014)	0.002 (0.014)	–0.014 (0.017)	–0.015 (0.017)	–0.013 (0.021)	–0.013 (0.021)
**Parental academic involvement (student report)**						
Homework help	0.006 (0.013)	0.002 (0.013)	–0.016 (0.017)	–0.017 (0.017)	–0.014 (0.020)	–0.016 (0.020)
Homework check	0.019 (0.013)	0.026* (0.013)	0.024 (0.016)	0.028 (0.016)	0.071*** (0.019)	0.065** (0.020)
Parental daily involvement (parent report)	–0.013 (0.021)	–0.012 (0.022)	0.010 (0.027)	0.013 (0.027)	0.051 (0.033)	0.039 (0.033)
Parental daily involvement (student report)	0.158*** (0.019)	0.159*** (0.019)	0.194*** (0.024)	0.195*** (0.024)	0.168*** (0.029)	0.164*** (0.029)
Parent–child communication (parent report)	0.026 (0.018)	0.013 (0.019)	0.012 (0.023)	0.001 (0.024)	0.011 (0.028)	0.024 (0.028)
Parent–child communication (student report)	0.143*** (0.016)	0.140*** (0.016)	0.234*** (0.021)	0.233*** (0.021)	0.304*** (0.025)	0.307*** (0.025)
**Parental school participation(parent report)**						
Help	0.025 (0.016)	0.022 (0.016)	0.015 (0.020)	0.014 (0.020)	0.043 (0.024)	0.038 (0.024)
Attend	–0.020 (0.018)	–0.013 (0.018)	–0.022 (0.023)	–0.017 (0.024)	–0.056* (0.028)	–0.054 (0.028)
**Parental school participation (student report)**						
Help	0.014 (0.015)	0.016 (0.015)	0.028 (0.019)	0.031 (0.019)	0.122*** (0.023)	0.115*** (0.023)
Attend	0.035* (0.016)	0.037* (0.016)	0.054** (0.020)	0.056** (0.021)	0.085*** (0.024)	0.090*** (0.024)
Female (vs. male)		0.071*** (0.020)		0.060* (0.025)		–0.113*** (0.030)
Only child (vs. more than one child)		0.042 (0.022)		–0.003 (0.028)		0.008 (0.033)
Han (vs. ethnic minorities)		–0.002 (0.054)		–0.039 (0.069)		–0.050 (0.082)
**Grade level (vs. Grade 9)**						
Grade 7		0.039 (0.026)		0.041 (0.033)		0.007 (0.039)
Grade 8		–0.002 (0.026)		0.020 (0.033)		–0.048 (0.039)
Family annual income (in USD)		0.005 (0.004)		0.003 (0.004)		0.006 (0.005)
Mother education level (vs. no undergraduate)		0.033 (0.037)		–0.013 (0.047)		0.077 (0.056)
Father education level (vs. no undergraduate)		–0.006 (0.036)		–0.021 (0.046)		–0.004 (0.055)
Adjusted *R*^2^	22.3%	22.8%	24.8%	24.8%	32.2%	32.6%

(1) B, unstandardized coefficients; (2) *p < 0.05, **p < 0.01, ***p < 0.001; (3) School fixed effects were included in all the models.

### Behavioral engagement

Taking account of the school fixed effects, Model 1’s results demonstrated that higher levels of children’s perception of parental daily involvement (*B* = 0.158, *p* < 0.05), of parent–child communication (*B* = 0.143, *p* < 0.05), and of Attend (belonging to parental school participation) (*B* = 0.035, *p* < 0.05) were associated with a higher level of behavioral engagement among students (Table 4). Because the three parental involvement variables were on the same scale, a comparison between them indicated that children’s perception of parental daily involvement and then children’s perception of parent–child communication tended to matter more on student behavioral engagement than children’s perception of Attend. Specifically, one unit increase in children’s perception of parental daily involvement, parent–child communication, and Attend were associated with 0.158, 0.143, and 0.035 units increase in behavioral engagement, respectively. In contrast, parents’ and children’s perceptions of parental academic involvement (Parent: *B*_Homework Help_ = -0.006, *p* > 0.05, *B*_Homework Check_ = 0.001, *p* > 0.05; Student: *B*_Homework Help_ = 0.006, *p* > 0.05, *B*_Homework Check_ = 0.019, *p* > 0.05), parents’ perception of parental daily involvement (*B* = –0.013, *p* > 0.05), parents’ perception of parent–child communication (*B* = 0.026, *p* > 0.05), parents’ perception of parental school participation (Help: *B* = 0.025, *p* > 0.05; Attend: *B* = –0.020, *p* > 0.05), and children’s perception of Help (*B* = 0.014, *p* > 0.05) were dissociated with student engagement. Model 1 in total explained 22.3% of the variance in student behavioral engagement.

Considering both the school fixed effects and the control variables (Model 2), children’s perception of parental daily involvement (*B* = 0.159, *p* < 0.05), children’s perception of parent–child communication (*B* = 0.140, *p* < 0.05), and children’s perception of Attend (*B* = 0.037, *p* < 0.05) were still positively associated with behavioral engagement (Table 4). Children’s perception of parental daily involvement and then children’s perception of parent–child communication continued to influence student behavioral engagement more than children’s perception of Attend. Children’s perception of Homework Check (belonging to parental academic involvement) became significantly and positively related to behavioral engagement, *B* = 0.026, *p* < 0.05. The other parental involvement variables were not associated with behavioral engagement when everything else being equal. Model 2 in total explained 22.8% of the variance in behavioral engagement, meaning that the control variables took account of 0.5% of the variance in behavioral engagement.

### Emotional engagement

After taking account of the school fixed effects (Model 1), children’s perception of parental daily involvement (*B* = 0.194, *p* < 0.05), children’s perception of parent–child communication (*B* = 0.234, *p* < 0.05), and children’s perception of Attend (belonging to parental school participation) (*B* = 0.054, *p* < 0.05) were positively related to student emotional engagement (see Table 4). A comparison between the three parental involvement variables indicated that children’s perception of parent–child communication and then children’s perception of parental daily involvement tended to matter more on student emotional engagement than children’s perception of Attend. More specifically, one unit increase in children’s perception of parent–child communication, parental daily involvement, and Attend were related to 0.234, 0.194, and 0.054 units increase in emotional engagement, respectively. The other parental involvement variables were not associated with emotional engagement. Model 1 in total explained 24.8% of the variance in student emotional engagement.

After taking account of both the school fixed effects and the control variables, children’s perception of parental daily involvement (*B* = 0.195, *p* < 0.05), children’s perception of parent–child communication (*B* = 0.233, *p* < 0.05), and children’s perception of Attend (*B* = 0.056, *p* < 0.05) remained to be associated with emotional engagement. The magnitudes of those associations were similar between Model 1 and Model 2 (see Table 4). Like Model 1, children’s perception of parent-child communication and then children’s perception of parental daily involvement mattered more than children’s perception of Attend on student emotional engagement. The other parental involvement variables were unrelated to emotional engagement. Model 2 explained 24.8% of the variance in student emotional engagement, suggesting that the control variables basically cannot explain any of the variances in emotional engagement.

#### Cognitive engagement

After considering the school fixed effects, children’s perception of Homework Check (belonging to parental academic involvement) (*B* = 0.071, *p* < 0.05), children’s perception of parental daily involvement (*B* = 0.168, *p* < 0.05), children’s perception of parent–child communication (*B* = 0.304, *p* < 0.05), and children’s perception of parental school participation (Help: *B* = 0.122, *p* < 0.05; *B* = 0.085, *p* < 0.05) were positively associated with students’ cognitive engagement (Table 4). A comparison between those parental involvement variables showed that children’s perception of parent–child communication had the strongest effect on student cognitive engagement, followed by children’s perception of parental daily involvement, children’s perception of parental school participation, and children’s perception of Homework Check. In contrast, parents’ perception of Attend (belonging to parental school participation) was negatively associated with student cognitive engagement, *B* = –0.056, *p* < 0.05. The other parental involvement variables did not exhibit significant relationships with student cognitive engagement. Model 1 in total explained 32.2% of the variance in student cognitive engagement.

Taking account of both the school fixed effects and the control variables, children’s perception of Homework Check (*B* = 0.065, *p* < 0.05), children’s perception of parental daily involvement (*B* = 0.164, *p* < 0.05), children’s perception of parent–child communication (*B* = 0.307, *p* < 0.05), and children’s perception of parental school participation (Help: *B* = 0.115, *p* < 0.05; *B* = 0.090, *p* < 0.05) remained positively associated with students’ cognitive engagement, and the magnitudes of the relationships were similar between Model 1 and Model 2 (Table 4). Similar to Model 1, children’s perception of parent–child communication had the strongest effect on student cognitive engagement, followed by children’s perception of parental daily involvement, children’s perception of parental school participation, and then children’s perception of Homework Check. Notably, once we added the control variables to the model, the negative association of parents’ perception of Attend with student cognitive engagement disappeared, *B* = –0.054, *p* > 0.05. The other parental involvement variables did not present statistically significant relationships with student cognitive engagement. Model 2 in total explained 32.6% of the variance in student cognitive engagement, denoting that the control variables altogether explained 0.4% of the variance in cognitive engagement.

#### A comparison among the models predicting behavioral engagement, emotional engagement, and cognitive engagement

The findings indicated that children’s perceptions of parental daily involvement, parent–child communication, and Attend (belonging to parental school participation) were positively associated with the three dimensions of student engagement. Among those three parental involvement variables, children’s perception of Attend had the weakest relationships with student engagement. Children’s perception of parental daily involvement had the strongest relationship with behavioral engagement, while children’s perception of parent–child communication had the strongest relationships with emotional engagement and cognitive engagement.

A comparison of the effects of parental involvement variables on student engagement indicated that the parental involvement variables had a stronger predictive ability on student cognitive engagement than on their behavioral engagement and emotional engagement. Ultimately, with all the other effects being controlled, none of the parental involvement variables from the parents’ perspectives was related to student engagement. In comparison, children’s perception of Homework Help (belonging to parental academic involvement) was the only parental involvement variable that was not associated with student engagement.

## Discussion and conclusion

### Discrepancies in parents’ and adolescents’ perceptions of parental involvement

Consistent with prior research (Paulson, 1994; Paulson and Sputa, 1996; DePlanty et al., 2007; Thomas et al., 2020; Liu et al., 2021), this study revealed that Chinese parents and their children had different perceptions of parental involvement. Some past studies found that parents reported higher levels of parental involvement behaviors than their children (Paulson, 1994; Paulson and Sputa, 1996; DePlanty et al., 2007), while the results showed that parents could either report a higher or a lower level of parental involvement than their children. Previous scholars drew contrasting conclusions, likely because they utilized different definitions of parental involvement behaviors and surveyed different student populations. For example, DePlanty et al. (2007) examined US rural junior high school students and their parents. In general, the findings of this study demonstrated that parents reported a higher tendency of checking their children’s homework and a higher level of parental daily involvement than those of their children’s perception. In contrast, parents reported a lower level of helping their children to complete homework, a lower level of parent–child communication, and a lower level of parental school participation. Notably, in this study, we considered parental homework help and homework check as reflective of parental academic involvement. Thus, the findings suggested that potential disparities existed between these two types of behaviors of parental academic involvement. Furthermore, because parental academic involvement, parental daily involvement, and parent–child communication measured parental home-based involvement, the findings suggested that perception disparities existed within home-based involvement. Because we only measured parental school-based involvement using parental school participation, we were not able to detect whether parents may report a lower level of other types of school-based involvement.

Interestingly, the findings of this study were not consistent with those of Liu et al. (2021) who also surveyed Chinese students and parents. They found that parents reported a higher level of parent–child communication and a lower level of parental academic involvement than their children. Because their research was carried out during the stay-at-home period during the COVID-19 pandemic, parents spent more time with their children all of a sudden and they felt obligated to communicate with their children more frequently. This might result in a higher level of parent–child communication perceived by parents than by their children. In addition, because parents experienced pressure from working at home and had to provide daily care to their children when confined at home (Fegert et al., 2020), they might have limited time to involve in their children’s academic activities. This fact might have explained the low level of parental academic involvement reported by parents than by children in Liu et al.’s (2021) study. The inconsistency between the findings of this study and the findings of Liu et al.’s (2021) study might also be due to the fact that we measured parental academic involvement differently in this study and distributed questionnaires during the post-pandemic period.

A comparison among all parental involvement variables indicated that both parents and children reported a higher level of parental daily involvement, but a relatively lower level of parental academic involvement, with parent–child communication and parental school participation in between. Similarly, although Liu et al. (2021) did not examine parental daily involvement, they found that both parents and children reported a higher level of parent-child communication than parental academic involvement. This might result from the relatively less time and energy required by the parental daily involvement than by the other types of parental involvement, especially parental academic involvement. Moreover, by inquiring Korean immigrant parents in the US, Kim et al. (2016) found that parents were inclined to view parental involvement as being pertinent to non-academic support at home (e.g., supporting children’s interests and communicating with children) than academic support, which explained the relatively lower level of parental academic involvement than, for example, parental daily involvement.

Nonetheless, the findings showed that all the parental involvement variables were positively associated with one another, and this implied that parents who were more academically involved in their children’s education tended to involve more in other types of parental involvement. For instance, a higher level of children’s perception of parental daily involvement was moderately and positively associated with a higher level of their perception of parent–child communication. In addition, we noticed that the magnitudes of the positive relationships among the parental involvement variables mostly ranged from none to weak. This not only exposed the existence of discrepancies in different types of parent involvement behaviors, but also presented the disparities in parents’ and children’s perceptions of parental involvement behaviors. Therefore, we maintained that while all the parental involvement variables did positively relate to one another, dissimilarities still existed. This finding corroborates the multifaceted nature of parental involvement (Fan and Chen, 2001). More importantly, we agreed with Korelitz and Garber (2016) to assert that the perception discrepancies between parents and children jeopardize the parent–child relationship. Future studies need to explore further the potential causes of the disparities among behaviors of parental involvement, by comparing and contrasting the perceptions of parents and their children.

### Relationships between parents’ and children’s perceptions of parental involvement and student engagement

The findings showed that different aspects of parental involvement presented varied and positive relationships with student behavioral, emotional, and cognitive engagement, which confirmed that parental involvement is not a unidimensional construct (Fan and Chen, 2001). More importantly, this study revealed that children’s perceptions of parental involvement behaviors, rather than those of their parents, were associated with student engagement. This is congruent with the study of Thomas et al. (2020), in which they found that children’s rather than parents’ perceptions of parental involvement were related to children’s achievement at school. This study, therefore, supports Epstein’s (1995) assertion that students act as the main actors in their education and development.

Consistent with previous research (e.g., Li and Lerner, 2013), the results of this study substantiated that behavioral engagement, emotional engagement, and cognitive engagement were positively associated with one another. Therefore, it is not surprising to find that the aspects of parental involvement that are related to one dimension of student engagement are also associated with other dimensions. However, the strength of these associations varied. Among the three significant parental involvement variables, children’s perception of parental daily involvement and closely followed by children’s perception of parent–child communication had the strongest relationship with student behavioral engagement; children’s perception of parent–child communication and closely followed by children’s perception of parental daily involvement had the strongest relationships with emotional engagement; children’s perception of parent-child communication had the strongest association with cognitive engagement; and, children’s perception of parental participation in school activities correlated with the three dimensions of student engagement to the weakest extent. Based on the ecological systems theory (Bronfenbrenner, 1979), parental daily involvement and parent-child communication are at the micro level in that parents directly interact with their children, which may explain why these two types of parental involvement had stronger relationships with student engagement than parental school participation. Future research, especially qualitative research, entails to explore the reasons behind the different relationships between parental involvement behaviors and the dimensions of student engagement.

In comparison, a higher frequency of parents checking their homework perceived by students was related to their behavioral engagement and cognitive engagement, but disconnected with their emotional engagement. This can be explained by the nature of behavioral engagement and cognitive engagement. That is, behavioral engagement refers to students’ positive conduct, involvement, and participation in academic-related activities; and cognitive engagement refers to students’ psychological and strategic investment in academic-related activities (Fredricks et al., 2004, 2016). In comparison, emotional engagement emphasizes on students’ emotional involvement toward school. Thus, having parents to check their children’s homework may encourage students, for example, to finish their homework ontime (behavioral engagement) as well as to do their schoolwork thoroughly and well rather than just trying to get by (cognitive engagement), while having parents to check homework may not lead students to feel the importance of schooling (emotional engagement).

A higher inclination of parents helping with organizing activities and events perceived by students was associated with their cognitive engagement, but not affecting their behavioral engagement and emotional engagement. It may be that parents who actively help their children’s school to organize activities and events have a closer connection with, for example, teachers whom may offer more suggestions and information to those parents. Considering the emphasis of Chinese parents on their children’s academic wellbeing (Kirkpatrick and Zang, 2011; Zhang, 2020), this may better and effectively guide those parents to provide assistance to their children’s learning, which may benefit students’ cognitive engagement.

Interestingly, from children’s perspectives, parental involvement in the form of offering help to their homework was not related to any dimensions of student engagement. The reasons may be twofold. First, the effect of parental involvement in offering help to homework on student engagement may be explained by the effects of other parental involvement variables. Second, parents who directly help their children to finish homework rather than to encourage their children to spend time and effort to figure out how to complete homework may not benefit student engagement. We encourage future qualitative research to investigate why certain parental involvement variables were related to the three dimensions of student engagement, while other variables were partially or not related to these dimensions.

With regard to practical implications, the findings of this study could help parents, teachers, and education practitioners to identify these parental involvement behaviors that may enhance student engagement in the long run. Analyzing the data from US high school students, Li and Lerner (2013) found that a higher level of behavioral engagement at an earlier grade was related to a higher level of student engagement at a later grade level. A higher level of emotional engagement at an earlier grade level was consistently related to a higher level of emotional engagement at a later grade. Meanwhile, a higher level of cognitive engagement at an earlier grade was consistently related to a higher level of cognitive engagement at a later grade. Converging the findings of this study with those of Li and Lerner (2013), it seems that a higher level of children’s perception of parental daily involvement, children’s perception of parent-child communication, and children’s perception of parental participation in school activities and events not only may predict a higher level of student engagement at present, but also may affect student engagement in the future. Meanwhile, children’s perceptions of parental homework check and of parental help in organizing school activities and events may have a larger impact on student cognitive engagement than on the other dimensions of student engagement for now and in the future. Ultimately, considering the cost-effectiveness and the rate of missing data, it might be more effective for future studies on parental involvement to collect data from children rather than from their parents.

## Limitations

This study has three main limitations. First, because parental academic involvement and parental school participation were measured by two items, respectively, it is difficult to compare them with other parental involvement variables. We did not create derived variables because the two items within these two parental involvement variables were not highly correlated with each other and because the preliminary analysis indicated that items measuring the two variables may present different relationships with student engagement. Second, in order to maintain the appropriate questionnaire length, we were not able to examine other types of parental involvement, such as parent-teacher communication in Liu et al.’s (2021) study. Third, since this is a cross-sectional study, we cannot claim any causal effects. Fourth, the nine middle schools under study were affiliated with a prestigious university and located in socioeconomically advantaged areas in China. This characteristic of the student sample may limit the generalizability of the findings to other poor and low-income populations inside and outside of China.

## Data availability statement

The original contributions presented in this study are included in the article/Supplementary material, further inquiries can be directed to the corresponding authors.

## Ethics statement

The studies involving human participants were reviewed and approved by the Academic Committee of Shandong University. Written informed consent to participate in this study was provided by the participants’ legal guardian/next of kin.

## Author contributions

KL, ML, and YZ designed the research. ML, YZ, and WL collected the data. KL did data analysis and wrote the manuscript. KL, ML, and YY reviewed the manuscript. All authors contributed to the article and approved the submitted version.

## References

[B1] Al-Alwan A. F. (2014). Modeling the relations among parental involvement, school engagement and academic performance of high school students. *Int. Educ. Stud.* 7 47–56. 10.5539/ies.v7n4p47

[B2] Ansong D. Okumu M. Bowen G. L. Walker A. M. Eisensmith S. R. (2017). The role of parent, classmate, and teacher support in student engagement: Evidence from Ghana. *Int. J. Educ. Dev.* 54 51–58. 10.1016/j.ijedudev.2017.03.010

[B3] Bong M. Woo Y. Shin J. (2013). Do students distinguish between different types of performance goals?. *J. Exp. Educ.* 81 464–489. 10.1080/00220973.2012.745464

[B4] Boonk L. Gijselaers H. J. M. Ritzen H. Brand-Gruwel S. (2018). A review of the relationship between parental involvement indicators and academic achievement. *Rev. Educ. Res.* 24 10–30. 10.1016/j.edurev.2018.02.001

[B5] Bronfenbrenner U. (1979). *The Ecology of Human Development. Experiments by Nature and Design.* Cambridge, MA: Harvard College.

[B6] Browne M. W. Cudeck R. (1993). “Alternative ways of assessing model fit,” in *Testing Structural Equation Models*, eds Bollen K. A. Long J. S. (Newbury Park, CA: Sage), 136–162.

[B7] Chowa G. A. N. Masa R. D. Tucker J. (2013). The effects of parental involvement on academic performance of Ghanaian youth: Testing measurement and relationships using structural equation modeling. *Child. Youth Serv. Rev.* 35 2020–2030. 10.1016/j.childyouth.2013.09.009

[B8] Connell J. P. Spencer M. B. Aber J. L. (1994). Educational risk and resilience in African-American youth: Context, self, action, and outcomes in school. *Child Dev.* 65 493–506. 10.2307/1131398 8013236

[B9] Dagnall N. Denovan A. Parker A. Drinkwater K. Walsh R. S. (2018). Confirmatory factor analysis of the inventory of personality organization-reality testing subscale. *Front. Psychol.* 9:1116. 10.3389/fpsyg.2018.01116 30026714 PMC6041939

[B10] Davidson T. M. Cardemil E. V. (2009). Parent-child communication and parental involvement in Latino adolescents. *J. Early Adolesc*. 29 99–121. 10.1177/0272431608324480

[B11] DePlanty J. Coulter-Kern R. Duchane K. A. (2007). Perceptions of parent involvement in academic achievement. *J. Educ. Res*. 100 361–368. 10.3200/JOER.100.6.361-368

[B12] Desimone L. (1999). Linking parent involvement with student achievement: Do race and income matter?. *J. Educ. Res*. 93 11–30. 10.1080/00220679909597625

[B13] Epstein J. L. (1995). School/family/community partnerships: Caring for the children we share. *Phi Delta Kappan* 76 701–712. 10.1177/003172171009200326

[B14] Erdem C. Kaya M. (2020). A meta-analysis of the effect of parental involvement on students’ academic achievement. *J. Learn. Dev.* 7 367–383. 10.1007/s10964-019-01072-5 31312979 PMC6732159

[B15] Fan W. Williams C. M. (2010). The effects of parental involvement on students’ academic self-efficacy, engagement and intrinsic motivation. *Educ. Psychol.* 3 53–74. 10.1080/01443410903353302

[B16] Fan X. Chen M. (2001). Parental involvement and students’ academic achievement: A meta-analysis. *Educ. Psychol. Rev*. 13 1–22. 10.1023/A:1009048817385

[B17] Fegert J. M. Vitiello B. Plener P. L. Clemens V. (2020). Challenges and burden of the Coronavirus 2019 (COVID-19) pandemic for child and adolescent mental health: A narrative review to highlight clinical and research needs in the acute phase and the long return to normality. *Child Adolesc. Psychiatry Ment. Health* 14:20. 10.1186/s13034-020-00329-3 32419840 PMC7216870

[B18] Finn J. D. Pannozzo G. M. Voelkl K. E. (1995). Disruptive and inattentive-withdrawn behavior and achievement among fourth graders. *Elem. Sch. J.* 95 421–434.

[B19] Finn J. D. Rock D. A. (1997). Academic success among students at risk for school failure. *J. Appl. Psychol.* 82 221–234. 10.1037/0021-9010.82.2.221 9109280

[B20] Finn J. D. Zimmer K. S. (2012). “Student engagement: What is it? Why does it matter?,” in *Handbook of research on student engagement*, eds Christenson S. L. Reschly A. L. Wylie C. (New York, NY: Springer), 97–131. 10.1007/978-1-4614-2018-7_5

[B21] Fredricks J. A. Blumenfeld P. C. Paris A. H. (2004). School engagement: Potential of the concept, state of the evidence. *Rev. Educ. Res.* 74 59–109. 10.3102/00346543074001059

[B22] Fredricks J. A. Filsecker M. Lawson M. A. (2016). Student engagement, context, and adjustment: Addressing definitional, measurement, and methodological issues. *Learn. Instr.* 43 1–4. 10.11124/jbisrir-2015-1919 26447007

[B23] Fu A. S. Markus H. R. (2014). My mother and me: Why tiger mothers motivate Asian Americans but not European Americans. *Pers. Soc. Psychol. Bull.* 40 739–749. 10.1177/0146167214524992 24727812

[B24] Hill N. E. Tyson D. F. (2009). Parental involvement in middle school: A meta-analytic assessment of the strategies that promote achievement. *Dev. Psychol.* 45 740–763. 10.1037/a0015362 19413429 PMC2782391

[B25] Jeynes W. H. (2007). The relationship between parental involvement and urban secondary school student academic achievement: A meta-analysis. *Urban Educ.* 42 82–110. 10.1177/0042085906293818

[B26] Kim Y. A. An S. Kim H. C. L. Kim J. (2016). Meaning of parental involvement among Korean immigrant parents: A mixed-methods approach. *J. Educ. Res.* 111 127–138. 10.1080/00220671.2016.1220355

[B27] Kirkpatrick R. Zang Y. (2011). The negative influences of exam-oriented education on Chinese high school students: Backwash from classroom to child. *Lang. Test. Asia* 1:36. 10.1186/2229-0443-1-3-36

[B28] Korelitz K. E. Garber J. (2016). Congruence of parents’ and children’s perceptions of parenting: A meta-analysis. *J. Youth Adolesc.* 45 1973–1995. 10.1007/s10964-016-0524-0 27380467 PMC5222679

[B29] Krohn M. D. Stern S. B. Thornberry T. P. Jang S. J. (1992). The measurement of family process variables: The effect of adolescent and parent perceptions of family life delinquent behavior. *J. Quant. Criminol.* 8 287–315. 10.1007/BF01064550

[B30] Lee J. (2014). The relationship between student engagement and academic performance: Is it a myth or reality?. *J. Educ. Res.* 107 177–185. 10.1080/00220671.2013.807491

[B31] Lei H. Cui Y. Zhou W. (2018). Relationships between student engagement and academic achievement: A meta-analysis. *Soc. Behav. Pers.* 46 517–528. 10.2224/sbp.7054

[B32] Li Y. Lerner R. M. (2013). Interrelations of behavioral, emotional, and cognitive school engagement in high school students. *J. Youth Adolesc.* 42 20–32. 10.1007/s10964-012-9857-5 23180069

[B33] Liu K. Yang Y. Li M. Li S. Sun K. Zhao Y. (2021). Parents’ and adolescents’ perceptions of parental involvement and their relationships with depression among Chinese middle school students during the COVID-19 pandemic. *Child. Youth Serv. Rev.* 129:106190. 10.1016/j.childyouth.2021.106190 34511675 PMC8421081

[B34] Lui P. P. Rollock D. (2013). Tiger mother: Popular and psychological scientific perspectives on Asian culture and parenting. *Am. J. Orthopsychiatry* 83 450–456. 10.1111/ajop.12043 24164517

[B35] Marshall I. A. Jackman G. (2015). Parental involvement, student active engagement and the ‘Secondary Slump’ phenomenon—Evidence from a three-year study in a Barbadian secondary school. *Int. Educ. Stud.* 8 84–96. 10.5539/ies.v8n7p84

[B36] National Bureau of Statistics of China, (2021). *Average wage of employed persons in urban units.* Available online at: https://data.stats.gov.cn/english/easyquery.htm?cn=C01 (accessed on Jan 24, 2022).

[B37] Nunez J. C. Regueiro B. Suarez N. Pineiro I. Rodicio M. L. Valle A. (2019). Student perception of teacher and parental involvement in homework and student engagement: The mediating role of motivation. *Front. Psychol.* 10:1384. 10.3389/fpsyg.2019.01384 31263441 PMC6584913

[B38] Nunez J. C. Suarez N. Rosario P. Vallejo G. Valle A. Epstein J. L. (2015). Relationships between perceived parental involvement in homework, student homework behaviors, and academic achievement: Differences among elementary, junior high, and high school students. *Metacogn. Learn*. 10 375–406. 10.1007/s11409-015-9135-5

[B39] Paulson S. E. (1994). Relations of parenting style and parental involvement with ninth-grade students’ achievement. *J. Early Adolesc.* 14 250–267. 10.1177/027243169401400208

[B40] Paulson S. E. Sputa C. L. (1996). Patterns of parenting during adolescence: Perceptions of adolescents and parents. *Adolescence* 31 369–381. 8726896

[B41] Salmela-Aro K. Tang X. Symonds J. Upadyaya K. (2021). Student engagement in adolescence: A scoping review of longitudinal studies 2010-2020. *J. Res. Adolesc.* 31 256–272. 10.1111/jora.12619 33991151

[B42] Schnitzler K. Holzberger D. Seidel T. (2020). All better than being disengaged: Student engagement patterns and their relations to academic self-concept and achievement. *Eur. J. Psychol. Educ.* 36 627–652. 10.1007/s10212-020-00500-6

[B43] Shernoff D. J. Kelly S. Tonks S. M. Anderson B. Cavanagh R. F. Sinha S. (2016). Student engagement as a function of environmental complexity in high school classrooms. *Learn. Instr.* 43 52–60. 10.1016/j.learninstruc.2015.12.003

[B44] Thomas V. Muls J. Backer F. D. Lombaerts K. (2020). Middle school student and parent perceptions of parental involvement: Unravelling the associations with school achievement and wellbeing. *Educ. Stud.* 46 404–421. 10.1080/03055698.2019.1590182

[B45] Trautwein U. Ludtke O. Schnyder I. (2006). Predicting homework effort: Support for a domain-specific, multilevel homework model. *J. Educ. Psychol.* 98 438–456. 10.1037/0022-0663.98.2.438

[B46] Voelkl K. E. (1996). Measuring students’ identification with school. *Educ. Psychol. Meas.* 56 760–770. 10.1177/0013164496056005003

[B47] Wang M. Sheikh-Khalil S. (2014). Does parental involvement matter for student achievement and mental health in high school?. *Child Dev.* 85 610–625. 10.1111/cdev.12153 24033259

[B48] Xie K. Vongkulluksn V. W. Lu L. Cheng S. (2020). A person-centered approach to examining high-school students’ motivation, engagement and academic performance. *Contemp. Educ. Psychol.* 62:101877. 10.1016/j.cedpsych.2020.101877

[B49] Xiong Y. Qin X. Wang Q. Ren P. (2021). Parental involvement in adolescents’ learning and academic achievement: Cross-lagged effect and mediation of academic engagement. *J. Youth Adolesc.* 50 1811–1823. 10.1007/s10964-021-01460-w 34117608

[B50] Yang Y. Liu K. Li M. Li S. (2021). Students’ affective engagement, parental involvement, and teacher support in emergency remote teaching during the COVID-19 pandemic: Evidence from a cross-sectional survey in China. *J. Res. Technol. Educ.* 54 S1–S13. 10.1080/15391523.2021.1922104

[B51] You S. Sharkey J. (2009). Testing a developmental–ecological model of student engagement: A multilevel latent growth curve analysis. *Educ. Psychol.* 29 659–684. 10.1080/01443410903206815

[B52] Zhang W. (2020). Shadow education in the service of tiger parenting: Strategies used by middle-class families in China. *Eur. J. Educ.* 55 388–404. 10.1111/ejed.12414

