# Supplementary material for: Parents’ perception or children’s perception? Parental involvement and student engagement in Chinese middle schools
Source: Front Psychol. 2022 Nov 17;13:977678. doi: 10.3389/fpsyg.2022.977678 (PMC9714349; doi:10.3389/fpsyg.2022.977678)
Supplement: Supplementary file 1 [file Data_Sheet_1.pdf]

Appendix A

Variable Description

Variable Name	Variable Description	Mean/%	Min	Max	SD	Cronbach's α
Student school engagement (student report)						
Behavioral engagement	A derived variable based on mean of 6 items, e.g., "during the past 2-3 months, I complete homework on time."	4.546	1.667	5	0.506	0.786
Emotional engagement	A derived variable based on mean of 5 items, e.g., "I think that school is important."	4.551	1	5	0.653	0.875
Cognitive engagement	A derived variable based on mean of 5 items, e.g., "I attempt to do my schoolwork thoroughly and well, rather than just trying to get by."	4.014	1	5	0.824	0.933
Parental academic involvement (parent report)						
Homework Help	"I help my child to do his/her homework."	2.880	1	4	0.871	
Homework Check	"I check my child's homework."	3.160	1	4	0.852	
Parental academic involvement (student report)						
Homework Help	"My parents help me to do my homework."	2.980	1	4	1.009	
Homework Check	"My parents check my homework."	3.050	1	4	1.024	
Parental daily involvement (parent report)	A derived variable based on mean of 3 items, e.g., "I support my child to participate in extracurricular activities."	3.545	1	4	0.503	0.859
Parental daily involvement (student report)	A derived variable based on mean of 3 items, e.g., "My parents support me to participate in extracurricular activities."	3.504	1	4	0.621	0.822
Parent-child communication (parent report)	A derived variable based on mean of 6 items, e.g., "My child talks about things that bother him/her."	3.146	1	4	0.616	0.923
Parent-child communication (student report)	A derived variable based on mean of 6 items, e.g., "I talk about things that bother me with my parents."	3.259	1	4	0.738	0.922
Parental school participation (parent report)						
Help	"I help to organize activities and events at my child's school"	3.140	1	4	0.833	
Attend	"I attend activities and events held by my child's school"	3.380	1	4	0.701	
Parental school participation (student report)						

Help	"My parents help to organize activities and events at my school"	3.240	1	4	0.888
Attend	"My parents attend activities and events held by my school"	3.440	1	4	0.805
Female (student report)	Students' gender (Reference: Male)	52.3%	0	1	
Only child (parent report)	Whether student is the only child. (Reference: More than one)	44.1%	0	1	
Han (student report)	Whether a student is ethnic majority. (Reference: Minorities)	96.8%	0	1	
Grade level (student report)	Students' grade level (Reference: Grade 9)				
Grade 7		39.5%	0	1	
Grade 8		31.1%	0	1	
Family annual income (parent report)	Family annual income (in USD)	3.406	0.11	30.93	3.151
Mother education level (student report)	whether a student's mother received postsecondary education or not.	66.3%	0	1	
Father education level (student report)	whether a student's father received postsecondary education or not.	68.9%	0	1	

Notes: Since both parents and their children completed the questionnaires online, this study was able to adopt the forced answering option (respondents could choose not to start the survey or drop out before completing it). This led to no missing values for all the questions.

Appendix B

Stepwise Multiple Linear Regressions with School Fixed Effects (Dummy Coded Parental Academic Involvement and Parental School Participation Variables)

	Behavioral Engagement		Emotional Engagement		Cognitive Engagement	
	Model 1<br>B (*se*)	Model 2<br>B (*se*)	Model 1<br>B (*se*)	Model 2<br>B (*se*)	Model 1<br>B (*se*)	Model 2<br>B (*se*)
Parental academic involvement (parent report)						
Homework Help	0.020	0.011	0.039	0.038	0.038	0.036
	(0.023)	(0.024)	(0.030)	(0.030)	(0.036)	(0.036)
Homework Check	-0.005	-0.006	-0.005	-0.007	-0.029	-0.027
	(0.027)	(0.027)	(0.034)	(0.034)	(0.041)	(0.041)
Parental academic involvement (student report)						
Homework Help	-0.005	-0.010	-0.067*	-0.069*	-0.040	-0.044
	(0.025)	(0.025)	(0.032)	(0.032)	(0.038)	(0.038)
Homework Check	0.015	0.025	0.059	0.065*	0.086*	0.070
	(0.025)	(0.026)	(0.032)	(0.033)	(0.039)	(0.039)
Parental daily involvement (parent report)	-0.010<br>(0.021)	-0.008<br>(0.021)	0.014<br>(0.027)	0.016<br>(0.027)	0.062<br>(0.033)	0.046<br>(0.033)
Parental daily involvement (student report)	0.168***<br>(0.019)	0.169***<br>(0.019)	0.209***<br>(0.024)	0.210***<br>(0.024)	0.198***<br>(0.029)	0.191***<br>(0.029)
Parent-child communication (parent report)	0.018<br>(0.018)	0.008<br>(0.018)	0.007<br>(0.023)	-0.001<br>(0.023)	-0.006<br>(0.028)	0.011<br>(0.028)
Parent-child communication (student report)	0.157***<br>(0.016)	0.155***<br>(0.016)	0.250***<br>(0.020)	0.249***<br>(0.021)	0.339***<br>(0.025)	0.342***<br>(0.025)
Parental school participation (parent report)						
Help	0.055*	0.054*	0.039	0.040	0.104*	0.093*
	(0.026)	(0.026)	(0.034)	(0.034)	(0.041)	(0.041)
Attend	-0.026	-0.019	-0.055	-0.049	-0.116*	-0.117*
	(0.036)	(0.036)	(0.045)	(0.046)	(0.055)	(0.055)
Parental school participation (student report)						
Help	0.018	0.022	0.021	0.026	0.162***	0.151**
	(0.028)	(0.028)	(0.036)	(0.036)	(0.044)	(0.043)
Attend	0.044	0.046	0.081*	0.083*	0.194***	0.200***
	(0.032)	(0.033)	(0.041)	(0.042)	(0.050)	(0.050)
Female (vs. male)		0.063**<br>(0.020)		0.052*<br>(0.025)		-0.137***<br>(0.030)
Only child (vs. more than one child)		0.042<br>(0.022)		-0.001<br>(0.028)		0.011<br>(0.034)
Han (vs. ethnic minorities)		0.000<br>(0.054)		-0.035<br>(0.069)		-0.040<br>(0.083)
Grade level (vs. Grade 9)						
Grade 7		0.039		0.040		0.013
		(0.026)		(0.033)		(0.040)
Grade 8		0.003		0.028		-0.037
		(0.026)		(0.033)		(0.040)
Family annual income (in USD)		0.005<br>(0.004)		0.004<br>(0.004)		0.006<br>(0.005)
Mother education level (vs. no undergraduate)		0.024<br>(0.037)		-0.017<br>(0.047)		0.051<br>(0.057)
Father education level (vs. no undergraduate)		-0.005<br>(0.037)		-0.013<br>(0.047)		0.005<br>(0.056)
Adjusted R^2	21.8%	22.2%	24.4%	24.4%	30.3%	30.9%

Notes: 1. B = Unstandardized coefficients; 2. * *p* < 0.05, ** *p* < 0.01, *** *p* < 0.001; 3. School fixed effects were included in all the models.
